# The microRNA miR-29a is associated with human immunodeficiency virus latency

**DOI:** 10.1186/s12977-014-0108-6

**Published:** 2014-12-09

**Authors:** Paresh Patel, Mohammad Yunus Ansari, Shraddha Bapat, Madhuri Thakar, Raman Gangakhedkar, Shahid Jameel

**Affiliations:** International Centre for Genetic Engineering and Biotechnology, New Delhi, 110067 India; National AIDS Research Institute, Pune, India; Current Address: The Wellcome Trust/DBT India Alliance, Plot No. 19, 8-2-684/3 K/19, Road No. 12, Banjara Hills, Hyderabad, 500034 India

**Keywords:** HIV-1, Latency, Viral replication, miRNA, miR-29a, Nef

## Abstract

**Background:**

Latent reservoirs of HIV-1 provide a major challenge to its cure. There are increasing reports of interplay between HIV-1 replication and host miRNAs. Several host miRNAs, which potentially target the *nef*-3′LTR region of HIV-1 RNA, including miR-29a, are proposed to promote latency.

**Findings:**

We used two established cellular models of HIV-1 latency – the U1 monocytic and J1.1 CD4+ T cell lines to show an inverse relationship between HIV-1 replication and miR-29a levels, which was mediated by the HIV-1 Nef protein. Using a miR-29a responsive luciferase reporter plasmid, an expression plasmid and an anti-miR29a LNA, we further demonstrate increased miR-29a levels during latency and reduced levels following active HIV replication. Finally, we show that miR-29a levels in the PBMCs and plasma of HIV infected persons also correlate inversely with latency and active viral replication.

**Conclusions:**

The levels of miR-29a correlate inversely with active HIV-1 replication in cell culture models and in HIV infected persons. This links miR-29a to viral latency and suggests another approach to activate and destroy latent HIV-1 reservoirs.

**Electronic supplementary material:**

The online version of this article (doi:10.1186/s12977-014-0108-6) contains supplementary material, which is available to authorized users.

## Findings

MicroRNAs (miRNAs) are 18–23 nucleotides non-coding, regulatory RNA molecules, which are involved in post-transcriptional gene regulation and have recently been shown to be important for regulating host responses in vertebrates [1]. During viral infections, miRNAs can either directly affect viral replication or modulate the expression of host genes and pathways essential for it [2]. For example, miR-32 and miR-181 restrict the replication of primate foamy virus 1 replication [3], and porcine reproductive and respiratory syndrome virus and Mink enteritis virus [4,5], respectively. Alternatively, miR-122 is highly expressed in the liver and facilitates hepatitis C virus replication [3,6]. The human immunodeficiency virus (HIV) may also be targeted by several host miRNAs [7]. The HIV-1 Tat and Nef proteins inhibit key components of the host miRNA pathway [8,9], knockdown of the miRNA biogenesis proteins Drosha and Dicer in latently infected cells increases HIV-1 replication, and host miRNAs such as the miR-17/92 cluster indirectly modulate HIV-1 replication through the p300/CBP-associated factor [10]. Thus, there is significant functional interplay between HIV-1 and miRNA-mediated silencing in host cells.

Following highly active antiretroviral therapy (HAART), the HIV-1 load is reduced dramatically to undetectable levels. However, replication competent viruses survive in latent reservoirs whose size is determined by the initial viral loads. On therapy interruption or failure, the virus replicates and repopulates peripheral sites [11]. The ability of HIV-1 to cause latent and persistent infection is a major impediment to its cure, making it important to understand the mechanisms of latency [12]. Profiling studies using various HIV-1 infection models have revealed different miRNAs associated with viral replication [13-16]. Although their contribution to HIV-1 latency is unclear, comparative miRNA expression in resting versus activated primary CD4+ T lymphocytes identified several host miRNAs, which potentially target the *nef*-3′UTR region to promote latency [17]. Several recent studies have shown that miR-29a, which targets the *nef*-3′UTR, is a potent inhibitor of HIV-1 replication [15,18,19]. Another report also showed miR-29a, −29b, −9 and -146a to target the SIV/HIV 3′UTR [20]. We hypothesize that miR-29a levels are high during latency and are reduced during active viral replication, and have explored this with cellular models of latent HIV-1 infection as well as PBMC and plasma from HIV infected individuals. All cell lines, other materials and methods used are detailed in Additional file 1: Materials and Methods.

**Figure 1:**
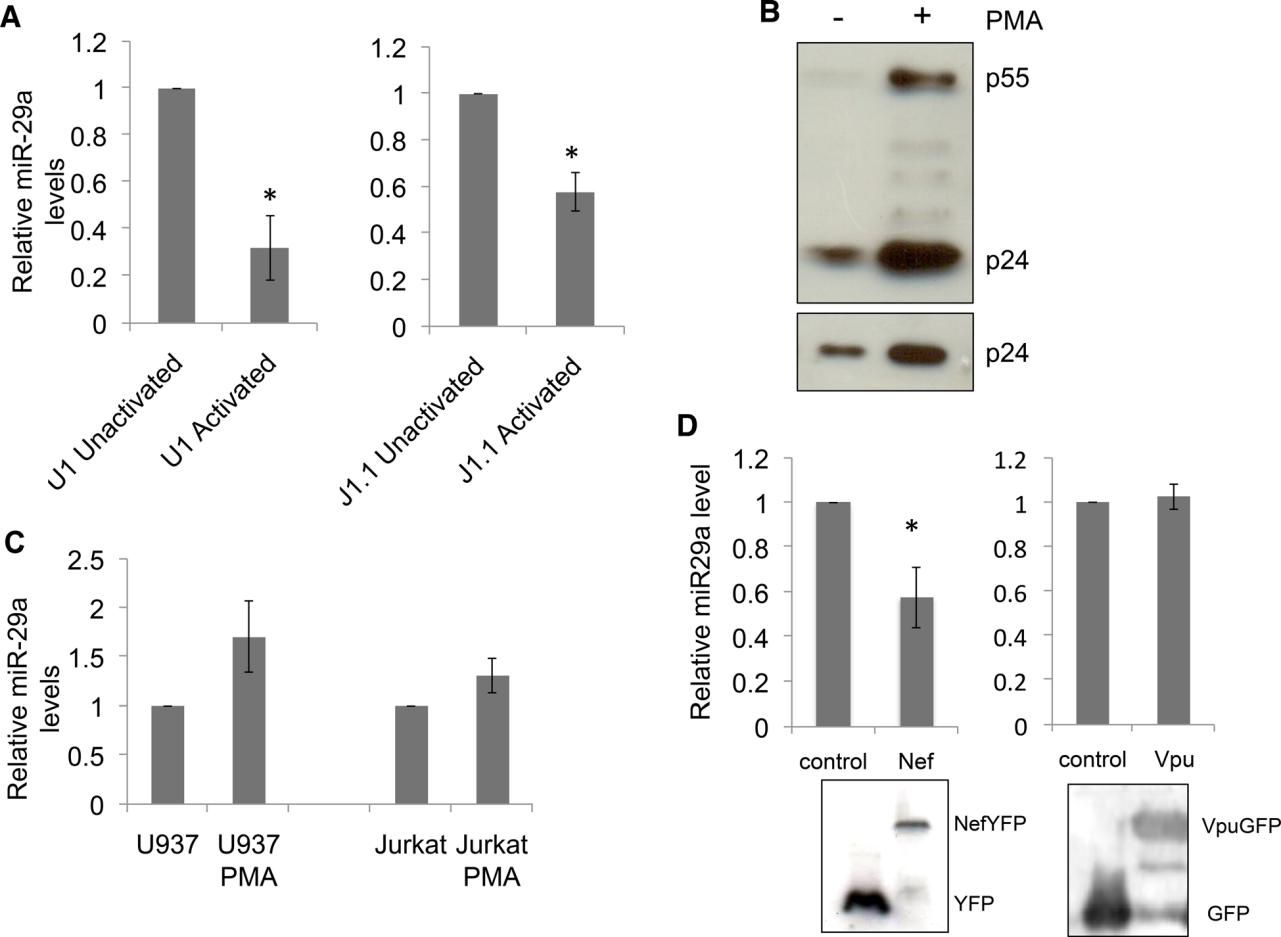
**MiR-29a levels are inversely correlated with activation in cellular models of HIV latency. (A)** The two cellular models of latency – U1 cells and J1.1 cells were activated with PMA, RNA was isolated and quantified for miR-29a levels as described in Materials and Methods. **(B)** U1 cells were treated with vehicle (DMSO) or PMA, and western blotting of cell lysates (upper panel) and culture supernatants (lower panel) was carried out with anti-p24 antibody. **(C)** U937 and Jurkat cell lines were activated with PMA, RNA was isolated and quantified for miR-29a levels as described in Materials and Methods. **(D)** U937 cells or stable U937 cell lines expressing either the HIV-1 Nef or Vpu protein were also used for quantifying miR-29a levels. Western blotting of the indicated cell lysates was carried out using anti-GFP antibodies. All data represents at least three independent experiments; *p < 0.05.

We used two well-established cell culture models of latent HIV-1 infection. These include U1, a derivative of U937 human monocytic cells and J1.1, derived from CD4^+^ Jurkat T cells [21,22]. Both these cell lines produce low basal levels of HIV-1, which is increased several folds on activation with phorbol myristic acid (PMA). If our hypothesis is correct, miR-29a levels should be high in the basal state when HIV-1 replication is low, but should decrease with PMA activation. Quantitative RT-PCR (qRT-PCR) showed this to be the case in U1 and J1.1 cells (Figure 1A). PMA activation also resulted in robust increase in virus production (Figure 1B). However, PMA activation of U937 or Jurkat cells did not reduce miR-29a levels (Figure 1C), suggesting that viral replication, but not activation *per se* is important for this effect. Being an early response, miR-29a levels may be modulated by a viral protein expressed early in the replication cycle. Since a large majority of early viral transcripts encode the Nef accessory protein [23], we quantified the levels of miR-29a in U937 cells that stably expressed this protein [24]. Compared to control cells, Nef-expressing U937 cells showed significantly reduced levels of miR-29a (Figure 1D). However, U937 cells stably expressing Vpu [25], an accessory protein expressed late in infection, showed no significant changes in miR-29a levels (Figure 1D).

**Figure 2:**
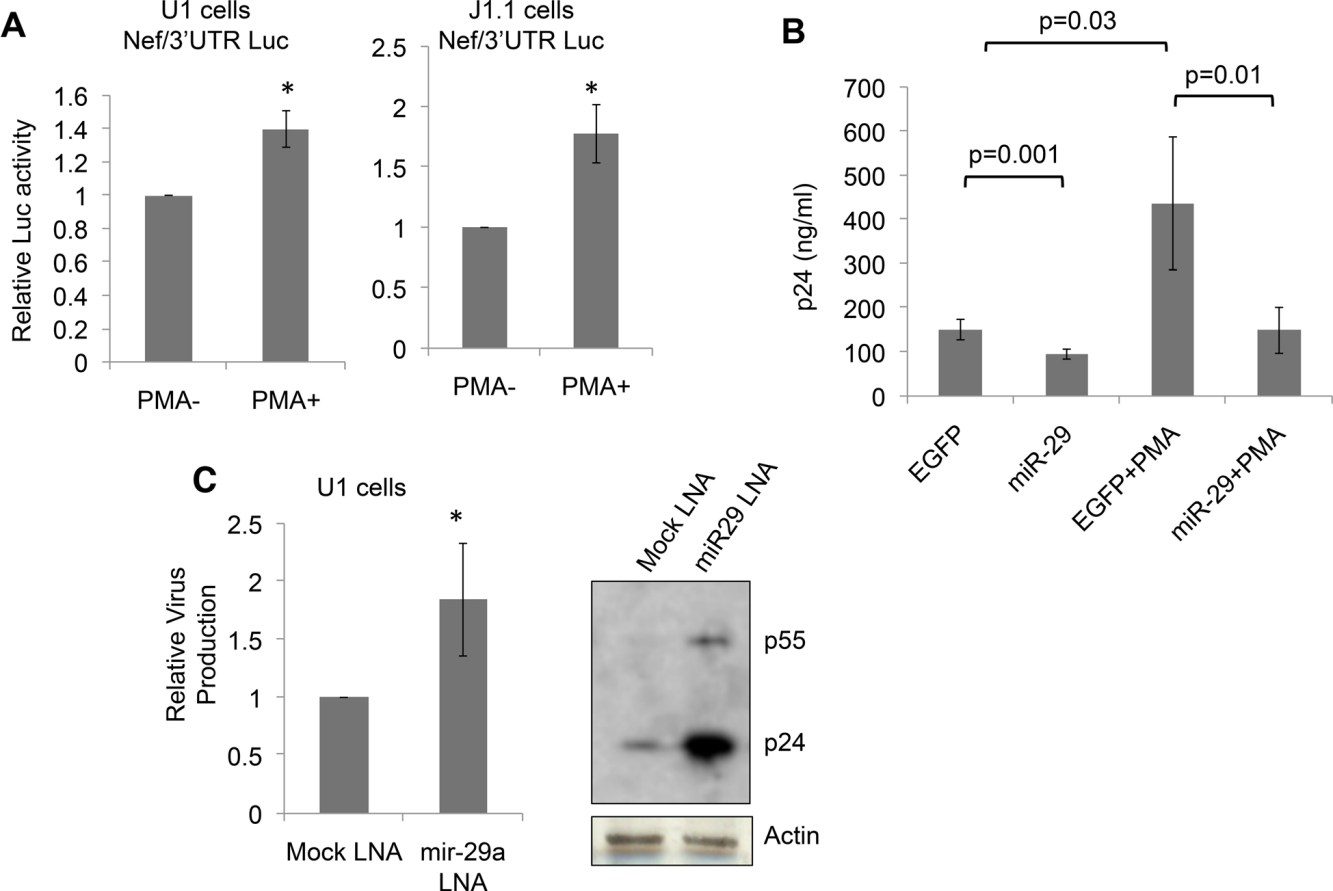
**Functional correlation between miR-29a levels and HIV replication. (A)** U1 cells and J1.1 cells were transfected with the pMIR-Report-Nef3′UTR reporter plasmid or the control pMIR-Report plasmid together with plasmid pRLTK. After 48 hr the cells were activated with PMA and the cell lysates quantified for luciferase activity as described in Materials and Methods. The luciferase activity in PMA activated cells is shown relative to unactivated cells after two normalizations - one with the Renilla luciferase activity to control for transfection efficiency and the other with luciferase activity observed with the control plasmid. Data represents three independent experiments; *p < 0.05. **(B)** J1.1 cells were transfected with the miR-29a-EGFP or EGFP expression vector. After 48 hr the cells were activated with PMA (or not) and HIV-1 production in culture supernatants was measured using a quantitative p24 ELISA. Transfection efficiency was around 30-40%. Data are shown as ng/ml of p24 and represent three independent experiments; the p-values for different sets are indicated. **(C)** U1 cells were transfected with a miR-29a specific LNA or a control LNA. After 48 hr HIV-1 production in culture supernatants was measured using a quantitative p24 ELISA. Data are shown relative to the EGFP control and represent three independent experiments; *p < 0.05. The western blot shows intracellular levels of Gag for the same cells; Actin was used as a loading control.

We then used the pMIR-Report-Nef3′UTR reporter plasmid, which contains the HIV-1 *nef*-3′UTR cloned downstream of the luciferase gene and is responsive to varying levels of miR-29a [18]. The pMIR-Report-Nef3′UTR or the control pMIR-Report constructs were transfected in U1 and J1.1 cells and the luciferase activities were measured. Following PMA activation, there was a significant increase in luciferase activity in pMIR-Report-Nef3′UTR transfected U1 and J1.1 cells (Figure 2A). This correlated with reduced miR-29a levels following HIV-1 activation. The J1.1 cells were then transfected with the pEGFP-miR-29a (or control pEGFP) expression plasmid and p24 levels were estimated in the culture medium. In miR-29a over-expressing J1.1 cells whether activated with PMA or not, p24 levels were reduced by ~60% compared to cells transfected with the control vector (Figure 2B). Finally, we reduced miR-29a levels in U1 cells with an anti-miR-29a LNA as described previously [18] and found increased virus levels in the culture supernatants and in cells (Figure 2C).

**Figure 3:**
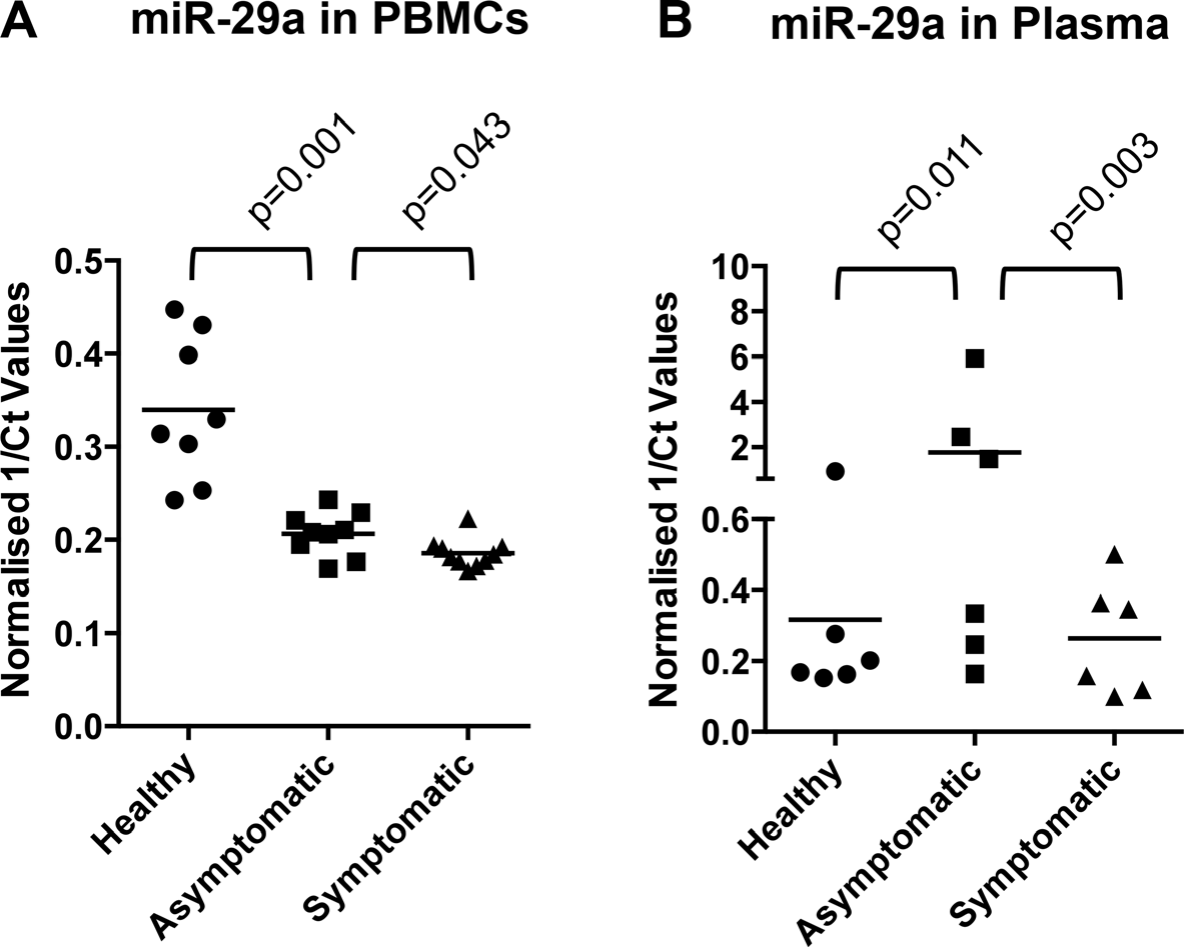
**MiR-29a levels in HIV-infected persons correlate with disease stage.** A cohort of HIV-infected persons was categorized into two groups – asymptomatic and symptomatic based on their CD4 counts. **(A)** PBMCs and **(B)** plasma from this cohort as well as healthy persons were quantified for miR-29a levels as described in Materials and Methods. The normalized 1/Ct values are plotted for each individual sample. The p values are shown.

In HIV infected but asymptomatic persons, the plasma viral load is detectable and absolute CD4 counts are high. In transition from the asymptomatic to symptomatic phase, HIV replicates actively leading to high viral loads and depletion of CD4^+^ T cells. In patients on antiretroviral therapy (ART), the plasma viral load goes down below detection limits (<50 copies of viral RNA per ml of plasma) but reservoirs harboring latent HIV continue to exist. In patients who discontinue ART or who fail on ART due to drug resistant mutations, the virus replicates rapidly leading to detectable plasma levels. We used a cohort of HIV-1 infected individuals who were categorized into either asymptomatic or symptomatic groups based on their CD4+ T cell counts, and quantified miR-29a levels in their PBMCs and plasma. The miR-29a levels were higher in PBMCs from asymptomatic persons in whom virus replication is restricted, compared to symptomatic patients in whom there is active viral replication (Figure 3A). This pattern of miR-29a expression was also observed in the plasma of asymptomatic and symptomatic patients (Figure 3B). We noted that miR-29a levels in PBMCs of healthy persons were higher than in HIV-infected persons, but the plasma levels of miR-29a showed an opposite trend (Figure 3A, B). Multiple tissues and cell types contribute the miR-29a in plasma, the regulation of which following HIV infection is poorly understood. We also observed recently that cellular levels of a given miRNA do not necessarily correlate with its secreted levels; some miRNAs are selectively retained in cells while others are preferentially secreted [26].

Previous studies on the role of miRNAs in HIV infection and pathogenesis have largely focused on acute infection models that involve actively replicating virus. Microarray analysis of acutely infected PBMCs showed downregulation of miR-29a and similar results were observed in PBMCs isolated from HIV infected individuals with high viral load [15,16]. These results directly support our hypothesis. In the U1 and J1.1 latency models we observed miR-29a levels to reduce following PMA activation and HIV-1 replication, and this correlated with expression of the Nef protein. Higher levels of miR-29a in the PBMCs and plasma of HIV-infected asymptomatic individuals compared to those with symptomatic disease, also support a role for miR-29a in HIV latency. Whether the size of the latent reservoir is determined early in infection or is related to peak viral loads or CD4/CD8 ratio is currently debatable. Our data support its relation to viral load, which is lower in asymptomatic individuals.

A functional miR-29a site is located in a highly conserved region within the HIV-1 *nef*-3′UTR. Previous studies have shown that co-transfection of HeLa cells with the HIV-1 pNL4-3 infectious clone and a miR-29a mimic leads to reduced p24 levels [15,18]. In physiologically relevant cell line models, we observed that overexpression of miR-29a further reduced HIV-1 replication, and a knockdown of miR-29a induced HIV-1 replication in latently infected cells without the need for PMA. Previous studies have shown that HIV-1 latency is controlled at the transcriptional level, and histone deacetylase (HDAC) inhibitors have been studied to break HIV-1 latency. Different HDAC inhibitors can induce HIV-1 replication from 2–20 folds in U1 cells [27]. Though the effects of blocking miR-29a on HIV-1 replication are small compared to those of HDAC inhibitors, these are still significant. Our findings suggest that HIV-1 latency may also be controlled at the post-transcriptional levels. This offers the opportunity for synergism with HDAC inhibitors in purging viral reservoirs.

The interaction of miR-29a and 3′UTR of HIV-1 RNA is sufficient for targeting the latter to the P bodies; the disruption of P bodies results in enhanced viral replication [19]. Host or environmental cues might release viral mRNAs from P bodies and thus release the suppression on viral replication [19]. Along these lines, increased miR-29a might target more viral mRNAs to P bodies and promote latency. The present study shows that HIV-1 Nef and miR-29a levels are inversely correlated, suggesting another mechanism for its activation of HIV-1 replication. Our data also suggest a role for miR-29a in maintaining HIV-1 latency and provides a new approach to activate (and destroy) latent HIV-1 reservoirs by inhibiting miR-29a. Further investigations are required to understand the tripartite regulatory axis of Nef, miR-29a and latency.

## Additional file

Additional file 1:
**Materials and Methods.**
