# Supplementary material for: The microRNA miR-29a is associated with human immunodeficiency virus latency
Source: Retrovirology. 2014 Dec 9;11:108. doi: 10.1186/s12977-014-0108-6 (PMC4269869; doi:10.1186/s12977-014-0108-6)
Supplement: Additional file 1: — Materials and Methods. [file 12977_2014_108_MOESM1_ESM.docx]

**Additional File**

**Materials and Methods**

***Cell lines.*** The HIV-1 latently infected cells, U1 and J1.1, were procured from the NIH AIDS Reagent Bank [21, 22]. The U937 stable cell lines expressing Nef and Vpu have been described elsewhere [24, 25]; these and their respective parental cell lines, U937 and Jurkat, were maintained in RPMI media supplemented with 10% fetal bovine serum (FBS).

***Patient samples.*** The PBMCs and plasma samples were obtained from HIV negative healthy individuals and HIV infected individuals at the National AIDS Research Institute (NARI), Pune, India after approval from the NARI Ethics Committee and written informed consent. Among the HIV infected individuals in different groups, those with HIV-2 and HIV-1/2 co-infection were excluded. The study only included adult participants, who were divided into four groups based on their CD4 counts and ART status, as follows: Asymptomatic, CD4 > 350 cells/μl and ART naïve; Symptomatic, CD4 < 450 cells/μl with opportunistic infection and ART naïve; On ART, CD4 < 350 cells/μl and receiving ART for at least 12 months; and ART Failure, those who satisfied WHO criteria for immunological, clinical or virological failure.

***RNA isolation and qRT-PCR.*** The U1, U937, J1.1 and Jurkat cells were cultured in fresh RPMI for 24 hours before RNA isolation. PBMCs were isolated by Ficoll-Paque for RNA isolation. For activation, U1 and J1.1 cells were treated with 50 ng/ml PMA for 48 hours. From cells, total RNA was isolated using the miRNAEasy kit (Qiagen, Gmbh) as per manufacturer’s protocol. The cDNA was prepared from 1 µg total cellular RNA using the mystiCq cDNA synthesis kit and the universal reverse primer (Sigma Aldrich). The real time PCR assay was performed on a StepOne Plus cycler (Life Technologies) using Evagreen (Solis-BioDine) and primers for hsa-miR-29a-3p and RNU6 (Sigma Aldrich). All the normalizations were done using RNU6 levels., Total RNA was isolated from 100 µl plasma using the miRNA Serum/Plasma Kit (Qiagen, Gmbh). The isolated plasma RNA was spiked with synthetic *C. elegans* miRNA mir-39 (cel-miR-39) for normalization. The RNA samples were diluted to 2 ng/µl and 5 µl of these diluted samples were used for cDNA synthesis using the kit described above for hsa-miR-29a-3p or specific SLRT primers for cel-mir-39. The PCR was performed using 1.25 ng of RNA equivalent as described above. All the normalizations for plasma samples were done using exogenously added cel-mir-39. For both cellular and plasma RNAs, the fold changes were calculated by the ∆∆CT method as described previously [17]. Statistical significance was calculated by paired two-tailed T Test and p<0.05 was considered significant.

***Nucleofection and reporter assay.*** The pMIR-Report-Nef3’UTR reporter plasmid, the control pMIR-Report plasmid and the miR-29a over expression construct, pEGFP-hsa-miR-29a, were a generous gift from Dr. Beena Pillai (Institute of Genomics and Integrative Biology, New Delhi, India) [18]. For nucleofection, 4 µg of plasmid was used with 2 million U1 or J1.1 cells and the Amexa Nucleofection Kit (Lonza). Cells were harvested 48 hours post-nucleofection. Plasmid pRLTK, which expresses the Renilla luciferase (1 µg), was also nucleofected into cells and used for normalization of transfection efficiency. The Dual-Glo luciferase reagent (Promega Corporation) was used to assay Firefly and Renilla luciferase as suggested by the manufacturer.

***p24 ELISA.*** The p24 levels in culture supernatants were estimated using a kit obtained from the NIH AIDS Reagent Bank as per manufacturer’s protocol.
